# Chronic Nitrous Oxide Exposure Disrupts Metabolism in Mice: A Plasma Untargeted Metabolomics Study

**DOI:** 10.3390/metabo16050324

**Published:** 2026-05-13

**Authors:** Juan Jia, Fenglin Zhang, Wen Zhang, Congying Liu, Keming Yun, Yujin Wang, Jiangwei Yan

**Affiliations:** 1 School of Forensic Medicine, Shanxi Medical University, Jinzhong 030600, China; jiajuan@sxmu.edu.cn (J.J.); 13920527687@163.com (W.Z.); 15757974250@163.com (C.L.); yunkeming5142@163.com (K.Y.); wangyj529@163.com (Y.W.); 2 Shanxi Key Laboratory of Forensic Medicine, Jinzhong 030600, China; 3 Shanxi Province Engineering Research Center of Forensic Identification, Jinzhong 030600, China; 4 Key Laboratory of Forensic Toxicology of Ministry of Public Security, Jinzhong 030600, China; 5 Jinan Public Security Bureau Zhangqiu Branch, Jinan 250200, China; zhangfenglin0131@163.com

**Keywords:** nitrous oxide (N_2_O), untargeted metabolomics, biomarkers, metabolic pathways, toxicity

## Abstract

Background: Nitrous oxide (N_2_O) is increasingly used as a recreational drug, leading to neurological and systemic toxicities. However, due to the rapid elimination and minimal alteration of nitrogen oxides, the short direct detection window complicates the assessment of N_2_O exposure. Method: In this study, we investigated the effects of chronic N_2_O exposure on plasma metabolites using an untargeted metabolomics approach in a mouse model. C57BL/6 mice were exposed to 90,000 ppm N_2_O (1 h, twice daily for 28 days) or room air. Plasma samples were analyzed via UHPLC -Triple TOF -MS. Orthogonal partial least squares discriminant analysis (OPLS-DA) and receiver operating characteristic (ROC) curves were used to identify differential metabolites. Result: A total of 35 differential metabolites were identified. Eight metabolites with an area under the curve (AUC) > 0.90 were selected as candidate biomarkers, including up-regulated SOPC and PC(16:0/16:0) (suggesting disrupted phospholipid remodeling and membrane integrity), and down-regulated DL-tryptophan, creatine, ectoine, indole, His-Ser, and Ile-Pro. Pathway enrichment analysis revealed significant alterations in glycine, serine and threonine metabolism; phenylalanine, tyrosine and tryptophan biosynthesis; protein digestion and absorption; and tryptophan metabolism. Conclusions: Our data indicate that chronic N_2_O exposure disrupts multiple amino acid-related metabolic pathways (e.g., tryptophan-kynurenine pathway) and phospholipid homeostasis. The identified metabolite changes, along with vitamin B_12_, homocysteine, and methylmalonic acid, may constitute a specific metabolic fingerprint for N_2_O exposure. These findings help reveal the intrinsic mechanistic links underlying metabolic disorders induced by N_2_O exposure.

## 1. Introduction

Nitrous oxide (N_2_O), commonly known as “laughing gas,” is a colorless, slightly sweet dissociative anesthetic gas. Since its discovery in the 18th century, it has had a long history of medical use in dentistry and labor analgesia [1,2]. However, in recent years, recreational abuse among adolescents has shown an increasing trend, drawing widespread concern [3]. Due to its ability to rapidly induce euphoria and sedation, coupled with ease of access and promotion via social media, the prevalence of recreational nitrous oxide use has increased steadily over the past decade. According to the Global Drug Survey (GDS), 22.5% of respondents reported lifetime N_2_O use, and nearly 10% reported past-year use. A seven-year trend analysis (2015–2021) revealed that global N_2_O use prevalence increased from 10% in 2015 to 20% in 2021 [4].

Recreational abuse of nitrous oxide can lead to serious adverse effects, including disorientation, psychomotor retardation, hypoxia, asphyxia, and myelopathy, posing a significant threat to both physical and mental health [5,6,7]. Acute poisoning can result from a single excessive inhalation, whereas chronic poisoning may follow long-term, high-dose abuse. Current research suggests that the underlying mechanism involves functional cobalamin deficiency, resulting from the oxidation and inactivation of cobalamin-dependent enzymes, particularly methionine synthase. Specifically, nitrous oxide irreversibly oxidizes the monovalent cobalt ion (Co^+^) of vitamin B_12_ to the inactive divalent (Co^2+^) and trivalent (Co^3+^) forms [8], thereby impairing the remethylation of homocysteine to methionine. This leads to downstream metabolic disturbances, including elevated homocysteine and methylmalonic acid (MMA) levels, which are clinical hallmarks of functional cobalamin deficiency. Consequently, even when circulating vitamin B_12_ levels appear normal, its metabolic function is compromised—a distinction crucial for accurate diagnosis and mechanistic understanding of nitrous oxide-induced neurological and hematological damage. Nevertheless, mounting evidence suggests that N_2_O may cause adverse health effects via other mechanisms, requiring thorough investigation [9].

As a gaseous substance, nitrous oxide (N_2_O) is minimally metabolized and is excreted predominantly in its unchanged form, primarily via the lungs. This results in rapid systemic elimination and a correspondingly short direct detection window [10]. Consequently, its metabolic parameters in the body remain largely unclear, and direct measurement of N_2_O itself is of limited utility for clinical or forensic detection. Current clinical detection of nitrous oxide abuse relies primarily on clinical symptoms and secondary biochemical indicators, including reduced plasma vitamin B_12_ levels and elevated homocysteine and methylmalonic acid (MMA) levels [11,12]. However, these indicators are surrogates of functional cobalamin deficiency rather than direct biomarkers of N_2_O exposure [13]. Importantly, they can be significantly influenced by multiple confounding variables, including dietary intake (e.g., vegetarianism), baseline vitamin B_12_ status, renal function (which affects MMA and homocysteine clearance), supplementation with B12 or folate, and the timing of blood sampling relative to N_2_O exposure [11,14]. Moreover, changes in these concentrations do not fully correspond to the extent or recency of nitrous oxide exposure. Therefore, these markers currently cannot provide a reliable research basis or objective evaluation criteria for clinical diagnosis or forensic identification, particularly in cases of single acute exposure or delayed presentation.

The application of metabolomics may offer a solution to this challenge. Metabolomics aims to comprehensively analyze concentrations of various metabolites and their dynamic changes in response to external stimuli. Untargeted metabolomics provides an effective means for discovering novel biomarkers or drug targets, exploring mechanisms of action, and conducting toxicological studies, making it an ideal method for metabolite discovery and hypothesis generation [15,16,17,18]. Recent advances in metabolomics and molecular profiling demonstrate that systemic biochemical changes can reveal disease mechanisms that traditional clinical biomarkers fail to capture [19,20]. For example, integrated transcriptomic–metabolomic analyses have been employed to elucidate therapeutic response metabolic pathways in neurological disease models, underscoring the value of multi-level molecular profiling in mechanistic interpretation [21]. Similarly, sensitive LC–MS/MS plasma analysis enables reliable detection of low-abundance metabolites and drug derivatives, establishing it as a powerful platform for toxicological and exposure-related investigations [22]. Meanwhile, recent mechanistic studies on proton-sensing and membrane-associated receptors highlight how subtle molecular perturbations translate into broader physiological responses [23].

This study aims to employ untargeted metabolomics to investigate alterations in endogenous small-molecule metabolites in the plasma of mice exposed to N_2_O, and to identify metabolic pathways closely associated with recreational nitrous oxide abuse through bioinformatics approaches. The goal is to explore the toxicological mechanisms of N_2_O abuse at the metabolic level, while identifying potential biomarkers, thereby providing new insights for the effective regulation of N_2_O and forensic identification in cases related to its abuse.

## 2. Materials and Methods

### 2.1. Chemicals and Reagents

N_2_O (99.9% purity) was provided by Henan Yuanzheng Specialty Gases Co., Ltd. (Henan, China). All solvents were of chromatographic grade and purchased from Fisher (Waltham, MA, USA). Ammonium acetate was obtained from Sigma (St. Louis, MO, USA).

### 2.2. Establishment of the Mouse Model

The experimental subjects were male C57BL/6 mice (n = 12) weighing approximately 20 g, purchased from Beijing Changyang Xishan Breeding Farm (Beijing, China) (License No. SCXK (Beijing) 2019-0010). These mice were randomly divided into a control group (n = 6) and a N_2_O exposure group (n = 6). Exposure experiments were conducted in a 60 L whole-body static chamber (with 6 mice per experimental session) under controlled environmental conditions with an ambient temperature of 22 ± 1 °C and a relative humidity of 50 ± 10%. Mice in the exposure group were subjected to 90,000 ppm N_2_O (volume fraction of 9%, mixed with ambient air; continuously monitored via an infrared gas analyzer) for 1 h per session, twice daily with a 4 h interval between sessions, over a consecutive 28-day period. During exposure, O_2_ concentration was maintained at 21 ± 0.5%, while CO_2_ level was kept below 0.3% (verified via random sampling). Control animals were housed in an adjacent chamber under identical conditions and exposed to ambient air equivalent to that in the exposure group. The N_2_O concentration of 90,000 ppm was selected based on a dose conversion extrapolated from a 60 kg adult human consuming 50 units of 8 g nitrous oxide cartridges per day, aiming to simulate chronic heavy recreational use while avoiding acute hypoxia [24]. During the 28-day exposure period, all mice were monitored daily for signs of acute toxicity, including changes in behavior (locomotor activity, grooming, rearing), alterations in respiratory rate, and the presence of seizures or loss of righting reflex. No overt signs of acute toxicity or behavioral abnormalities were observed in either the control or N_2_O-exposed groups. Body weights were recorded every three days, and no significant differences were found between the two groups throughout the experiment.

Twenty-four hours after the final exposure, orbital blood samples were collected and placed into pre-chilled (4 °C) heparin-containing Eppendorf tubes. After gentle mixing, place the mixture in a 4 °C centrifuge, then centrifuged at 1500 *g* for 10 min at 4 °C. The supernatant (approximately 100 μL) was aliquoted into pre-chilled (4 °C) Eppendorf tubes, rapidly frozen in liquid nitrogen, and stored at −80 °C until analysis. All experimental procedures were reviewed and approved by the Ethical Committee for Use of Laboratory Animals of the Shanxi Medical University (SYDL2025013).

### 2.3. Sample Preparation and Extraction

An appropriate quantity of each sample was thawed gradually in a refrigerator at 4 °C. Subsequently, a pre-cooled extraction solvent consisting of methanol/acetonitrile/water (2:2:1, *v*/*v*/*v*) was added to the samples, followed by vigorous vortexing to achieve homogeneous mixing. The resultant mixture was subjected to ultrasonic extraction in an ice bath for 30 min, incubated at −20 °C for 10 min, and then centrifuged at 14,000 *g* for 20 min at 4 °C. The collected supernatant was transferred and concentrated to dryness using vacuum evaporation. The dried residue was reconstituted with 100 μL of acetonitrile/water solution (1:1, *v*/*v*), homogenized by vortexing, and re-centrifuged at 14,000 *g* for 15 min at 4 °C. The final supernatant was filtered through a 0.22 μm microporous membrane and transferred into an autosampler vial for subsequent instrumental analysis. Quality control (QC) samples were prepared by pooling equal volumes of all individual test samples. These QC aliquots were randomly interspersed throughout the entire analytical run to monitor and evaluate the stability and reproducibility of the instrument performance.

### 2.4. UPLC Conditions

Chromatographic separation of all test samples was performed using an Agilent 1290 Infinity ultra-high-performance liquid chromatography system (Agilent Technologies, Santa Clara, CA, USA) equipped with a hydrophilic interaction liquid chromatography (HILIC) column (ACQUITY UPLC BEH Amide 1.7 μm, 2.1 mm × 100 mm column; Waters, Milford, MA, USA). The analytical conditions were set as follows: column temperature, 25 °C; flow rate, 0.5 mL/min; and injection volume, 2 μL. The mobile phase consisted of solvent A (ultrapure water containing 25 mM ammonium acetate and 25 mM ammonia solution) and solvent B (acetonitrile). The gradient elution procedure was programmed as follows: 0–0.5 min, 95% B; 0.5–7 min, B linearly decreased from 95% to 65%; 7–8 min, B linearly decreased from 65% to 40%; 8–9 min, 40% B held; 9–9.1 min, B linearly increased from 40% to 95%; 9.1–12 min, 95% B held. During the entire analytical run, all samples were preserved in an autosampler maintained at 4 °C. The samples were injected in a randomized order, with QC samples interspersed to minimize the impact of signal drift during instrument operation.

### 2.5. MS Conditions

After separation by the LC system, the samples were subjected to mass spectrometry analysis using an AB SCIEX Triple TOF 6600 (AB SCIEX, Framingham, MA, USA) mass spectrometer, operated in both positive and negative electrospray ionization (ESI) modes. The ESI source parameters were set as follows: nebulizer gas/auxiliary heating gas 1 (Gas1): 60 psi; auxiliary heating gas 2 (Gas2): 60 psi; curtain gas (CUR): 30 psi; ion source temperature: 600 °C spray voltage (ISVF): ±5500 V (for both positive and negative modes). The precursor ion scan range was m/z 60–1000 Da, and the product ion scan range was m/z 25–1000 Da. For MS/MS acquisition, the data-dependent acquisition (IDA) mode was employed, with selection based on peak intensity.

### 2.6. Processing and Statistical Analysis

The raw data were converted to appropriate formats, followed by peak alignment, retention time correction, and peak area extraction. Compound identification was performed on the XCMS 4.4.0 (Scripps, La Jolla, CA, USA) software output to confirm metabolite names. Subsequently, data preprocessing was conducted using the k-nearest neighbor (kNN) method for missing value imputation and sum normalization. Finally, quality assessment and data analysis were performed on the preprocessed experimental data. The identified metabolites were then subjected to class determination, quantitative analysis, and chemical classification. Intergroup differences were analyzed using statistical methods. In the OPLS-DA model, differential metabolites were screened using strict criteria of variable importance for the projection (VIP) > 1 and a *t*-test *p*-value < 0.05, and potential biomarkers were identified. The implicated pathways of biomarkers were interpreted using the KEGG database.

## 3. Results

### 3.1. Stability and Reliability Analysis of Detection Method

**Figure 1:**
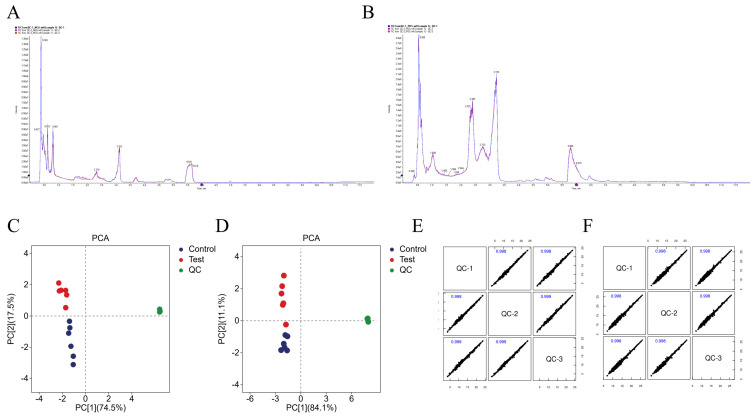
Analysis of the Stability and Accuracy of the Assay Method. (**A**,**B**) TIC Plot for Positive and Negative Ion Modes ((**A**) positive ion mode; (**B**) negative ion mode); (**C**,**D**) PCA Plot for Positive and Negative Ion Modes ((**C**) positive ion mode; (**D**) negative ion mode); (**E**,**F**) Correlation map of quality control samples ((**E**) positive ion mode; (**F**) negative ion mode).

In this experiment, principal component analysis (PCA) was performed on the peaks extracted from the experimental and quality control (QC) samples. The results showed that QC samples clustered tightly in both positive and negative ion modes, indicating good experimental reproducibility (Figure 1A–D). Pearson correlation analysis of the QC samples revealed correlation coefficients greater than 0.9 in both positive and negative ion modes, indicating high experimental reproducibility (Figure 1E,F).

### 3.2. Stability and Applicability Analysis of Model

**Figure 2:**
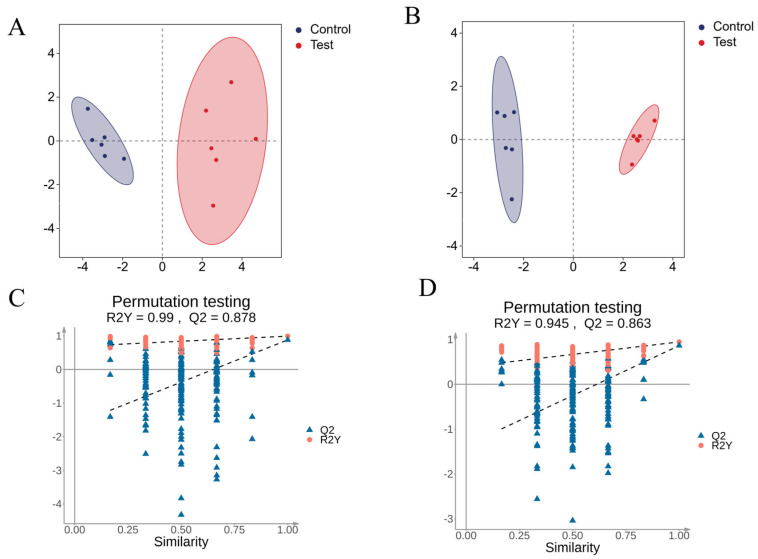
Analysis of Model Stability and Applicability. (**A**,**B**) OPLS-DA Score map of plasma ((**A**): positive ion mode; (**B**): negative ion mode); (**C**,**D**) OPLS-DA model permutation test of plasma ((**C**): positive ion mode; (**D**): negative ion mode).

Orthogonal partial least squares discriminant analysis (OPLS-DA) was performed for statistical analysis, which reflects intergroup variability and intra-group similarity (Figure 2A,B). The clear separation between the two groups was achieved, indicating that the metabolites in the experimental group mice differed from those in the control group. The validity of the model was assessed using a permutation test. The progressive decline in both R^2^ and Q^2^ of the random models with decreasing permutation retention demonstrated that the original model was not overfitted and was valid (Figure 2C,D).

### 3.3. Untargeted Metabolomics Analysis of Mice

**Figure 3:**
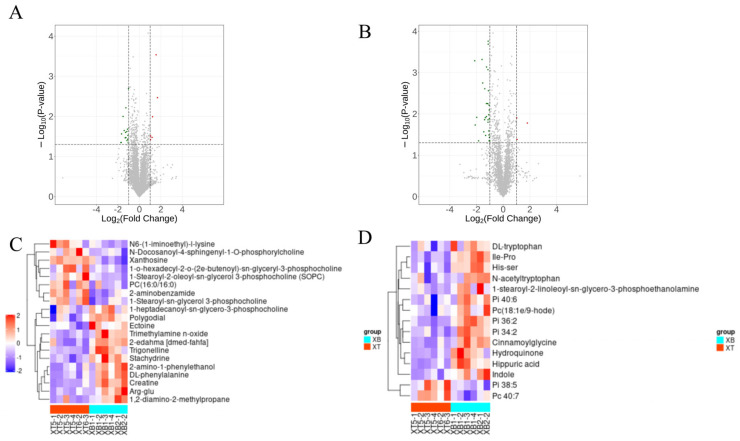
Changes in plasma metabolite profiles between the control and experimental groups of mice. (**A**,**B**) Volcano plot of fold change analysis in plasma samples ((**A**): positive ion mode; (**B**): negative ion mode); (**C**,**D**) heat map of plasma differential metabolites ((**A**): positive ion mode; (**B**): negative ion mode).

A total of 921 metabolites were identified in the plasma samples, of which 569 were detected in positive ion mode and 352 in negative ion mode. According to the chemical classification of the identified metabolites, lipids and lipid-like molecules, as well as organic acids and their derivatives, were the predominant categories. Using variable importance for the projection (VIP) > 1, *t*-test *p* < 0.05 and |log2FC| > 1 as criteria for significant differential metabolites, a total of 35 differential metabolites were identified in this study (Table 1, Figure 3A,B). Compared with the control group, 14 metabolites were significantly up-regulated and 21 metabolites were significantly down-regulated in the exposure group. Heat map visualization of the differential metabolites demonstrated marked differences between the N_2_O exposure group and the control group (Figure 3C,D).

**Figure 4:**
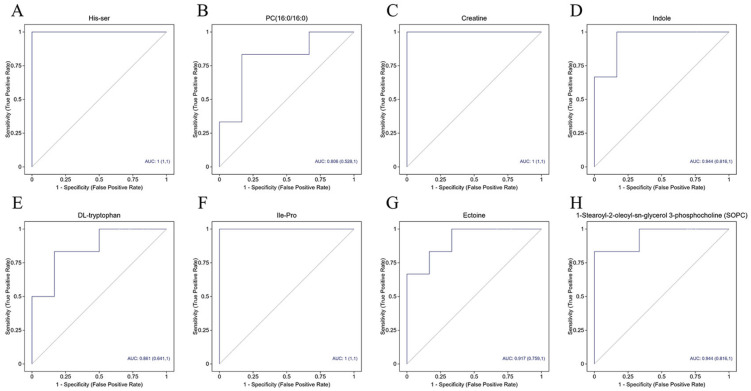
ROC curves of plasma differential metabolite. (**A**) His-ser; (**B**) PC(16:0/16:0); (**C**) Creatine; (**D**) Indole; (**E**) DL-tryptophan; (**F**) Ile-Pro; (**G**) Ectoine; (**H**) 1-Stearoy1-2-oleoy1-sn-glycerol-3-phosphocholine (SOPC).

The clinical diagnostic value of the differential metabolites identified between the exposure group and the control group was evaluated using the area under the ROC curve (AUC). (Figure 4) Metabolites with an AUC value > 0.90 were, as speculated, related metabolites. The results showed that among the 35 differential metabolites, eight biomarkers—including DL-tryptophan, creatine, ectoine, and indole—exhibited favorable AUC values and could serve as potential biomarkers for the diagnosis of chronic nitrous oxide poisoning.

### 3.4. Enrichment Analysis of Metabolic Pathways for Differential Markers

**Figure 5:**
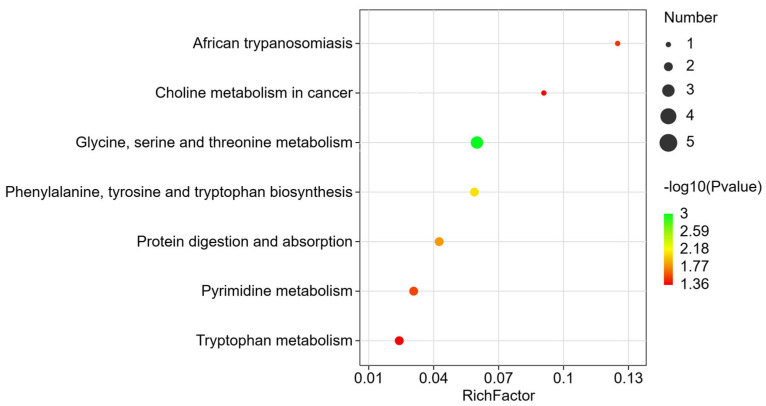
Differential metabolite pathway enrichment analysis bubble map in plasma.

Through metabolic pathway enrichment analysis using the KEGG database, seven metabolic pathways were identified as significantly different from those in the control group: glycine, serine and threonine metabolism; phenylalanine, tyrosine and tryptophan biosynthesis; protein digestion and absorption; pyrimidine metabolism; African trypanosomiasis; tryptophan metabolism; and choline metabolism in cancer. (Figure 5) These seven significantly altered metabolic pathways primarily involved eight differential metabolites. Among them, 1-stearoyl-2-oleoyl-sn-glycero-3-phosphocholine (SOPC) and 1,2-dipalmitoyl-sn-glycero-3-phosphocholine (PC(16:0/16:0)) were up-regulated, whereas DL-tryptophan, creatine, ectoine, indole, His-Ser, and Ile-Pro were down-regulated, thereby affecting multiple metabolic pathways in mice.

## 4. Discussion

The current situation around nitrous oxide abuse is concerning. Accumulating evidence indicates that long-term abuse of nitrous oxide exerts adverse effects on human health, underscoring the urgent need for in-depth investigation [5]. This study used untargeted metabolomics to investigate the metabolomic differences between nitrous oxide-intoxicated mice and controls. By combining differential metabolite screening with receiver operating characteristic (ROC) analysis, this study identified eight biomarkers with significant clinical application potential (*p* < 0.05, AUC > 0.90).

Amino acids are essential organic compounds for human growth and development, serving both as key substrates for protein synthesis and as the material foundation for metabolism and physiological functions. Our study identified that differential metabolites such as tryptophan, indole, and glycine participate in the body’s regulation following nitrous oxide abuse.

Tryptophan is an essential amino acid for humans. Most of it is metabolized via the kynurenine pathway into metabolites such as kynurenic acid and xanthurenic acid, which are involved in the regulation of inflammation and the immune system. On one hand, its metabolites exert neurotoxicity by promoting glutamate release from neurons and inhibiting its cellular reuptake, thereby further elevating extracellular glutamate levels and activating the glutamatergic system [25]. On the other hand, these metabolites can scavenge oxygen free radicals and exhibit antioxidant activity, thereby exerting neuroprotective effects. A small portion of tryptophan is enzymatically converted to serotonin (5-HT), which acts as a neurotransmitter involved in central nervous system regulation [26,27]. In this study, compared with the control group, the N_2_O-exposed group exhibited reduced DL-tryptophan levels. Tryptophan-related metabolism may be disrupted, thereby affecting the regulation of the nervous system. This could explain the neurological dysfunction (such as paresthesia and short-term memory impairment) observed in individuals with nitric oxide abuse.

Indole is an important metabolite in the tryptophan indole metabolic pathway. It acts as an intercellular molecular signal involved in host protection and defense against pathogens. Additionally, indole can activate the aryl hydrocarbon receptor (AhR) to modulate immune responses, thereby further regulating intestinal mucosal immunity, the gut barrier, and intestinal homeostasis [28]. In this study, indole levels were decreased in the N_2_O-exposed group compared with controls. The observed decrease in indole suggests a potential disruption of tryptophan indole metabolic flux, which may affect three pathways: phenylalanine, tyrosine and tryptophan biosynthesis; protein digestion and absorption; and tryptophan metabolism. It is therefore plausible—though not directly tested here—that reduced indole could contribute to intestinal immune imbalance or dysbiosis, potentially relating to gastrointestinal symptoms (e.g., nausea) reported in N_2_O abusers. However, direct measurements of gut microbiota, AhR activation, cytokines, or intestinal barrier integrity are needed to validate this hypothesis.

Glycine is a natural amino acid that plays important protective roles in neurological function and immune regulation [29,30]. In this study, creatine levels were decreased in the N_2_O-exposed group compared with controls. Creatine synthesis is dependent on glycine, and both are involved in energy metabolism and muscle function [31,32,33]. Pathway analysis identified the glycine, serine, and threonine metabolic pathway as enriched. Creatine, not glycine itself, was the measured metabolite showing a significant change. The observed decrease in creatine suggests a possible disturbance in energy metabolism during chronic N_2_O exposure, which may contribute to fatigue or muscle-related symptoms.

Ectoine, an amino acid derivative, exhibits dual anti-inflammatory and immunosuppressive activities on peripheral nerves and provides varying degrees of protection to certain biomacromolecules. It not only balances cellular osmotic pressure and maintains cellular homeostasis [34] but also protects unstable enzymes and DNA activity [35]. Thymine and deoxyuridine are involved in DNA synthesis [36]. In the present study, compared with the control group, decreased levels of ectoine were observed in the N_2_O-exposed group. Its involvement in the glycine, serine and threonine metabolic pathway may reduce the protective effect on DNA polymerase, leading to impaired DNA synthesis. Additionally, decreased levels of the dipeptides His-Ser and Ile-Pro may cause pyrimidine metabolic disturbance and consequently block DNA synthesis.

In this study, levels of 1-stearoyl-2-oleoyl-sn-glycero-3-phosphocholine (SOPC) and phosphatidylcholine (16:0/16:0) were significantly elevated in the N_2_O-exposed group compared with the control group. Phosphatidylcholines (PCs) are major components of cell membranes, pulmonary surfactant, and myelin sheaths [37]. Several mechanisms may explain this elevation. N_2_O induces functional vitamin B12 deficiency, which inactivates methionine synthase, impairs methionine synthesis, and reduces production of S-adenosylmethionine (SAM)—a universal methyl donor essential for PC synthesis via the CDP-choline pathway; thus, disruption of methylation homeostasis may lead to dysregulated PC metabolism. Additionally, chronic inhalation of exogenous gases can trigger pulmonary membrane remodeling and surfactant turnover, potentially facilitating PC release into the bloodstream. Given the established association between N_2_O abuse and myelopathy, elevated plasma PC levels may also reflect myelin lipid turnover or structural damage. Furthermore, alterations in lipoprotein-mediated lipid transport could contribute to PC accumulation in blood [38]. In summary, elevated SOPC and PC(16:0/16:0) may serve as metabolic signatures of long-term N_2_O exposure, and this change is likely closely associated with vitamin B_12_-dependent methylation dysfunction, pulmonary membrane remodeling, and myelin-related physiological processes.

**Figure 6:**
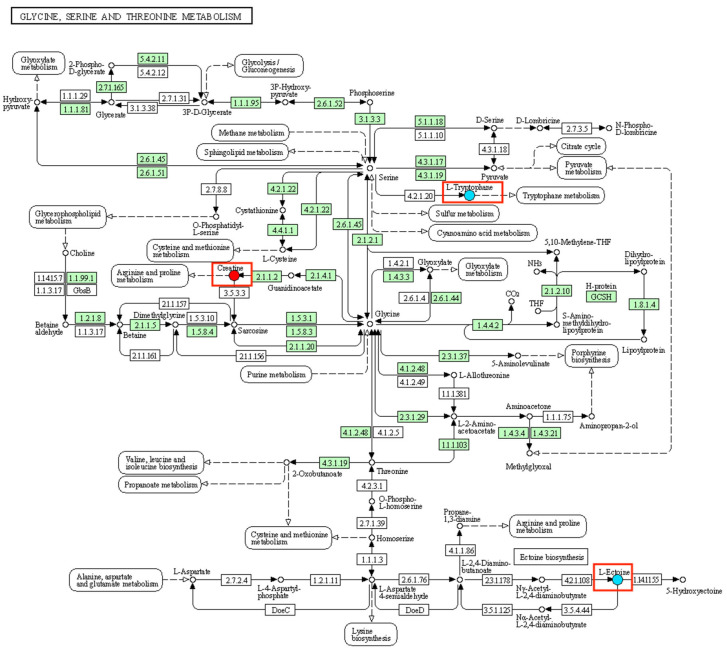
KEGG metabolic pathway map of glycine, serine, and threonine metabolism. Red squared parts highlight the positions of key differential metabolites (DL-tryptophan, creatine, and ectoine).

Meanwhile, vitamin B_12_ is an essential cofactor for methionine regeneration and tetrahydrofolate formation and is involved in the biosynthetic pathways of nucleic acid and amino acid metabolism [39]. Considering the clinical mechanisms of neurological damage associated with nitrous oxide abuse, along with the roles of the differential metabolites identified in this study that are related to the significantly altered metabolic pathways, the downregulation of DL-tryptophan, creatine, and ectoine is involved in amino acid metabolism and DNA synthesis, thereby playing important physiological roles in maintaining nutrition, regulating apoptosis, and supporting other vital functions (Figure 6). The above analyses suggest that 1-stearoyl-2-oleoyl-sn-glycero-3-phosphocholine (SOPC), PC(16:0/16:0), DL-tryptophan, creatine, and ectoine, together with the commonly used markers vitamin B_12_, homocysteine, and methylmalonic acid (MMA), may constitute a specific metabolic fingerprint of N_2_O exposure.

Collectively, this study reveals that chronic N_2_O exposure disrupts multiple metabolic pathways in mouse plasma, shedding light on the toxicological mechanisms underlying N_2_O intoxication, and the candidate biomarkers identified may facilitate the development of diagnostic and forensic approaches for N_2_O abuse.

## 5. Conclusions

N_2_O abuse affects multiple metabolic pathways, disrupting the normal metabolic profile of mice. It impacts pathways related to amino acid metabolism, including glycine, serine and threonine metabolism; phenylalanine, tyrosine and tryptophan biosynthesis; protein digestion and absorption; African trypanosomiasis and tryptophan metabolism. Based on the identification of significant differential metabolites with AUC values > 0.90 and the known mechanisms of neurological damage associated with N_2_O abuse, it is inferred that 1-stearoyl-2-oleoyl-sn-glycero-3-phosphocholine (SOPC), PC(16:0/16:0), DL-tryptophan, creatine, and ectoine, together with vitamin B_12_, homocysteine, and methylmalonic acid (MMA), may constitute characteristic biomarkers of N_2_O exposure.

Although the present study characterizes the plasma metabolic alterations following subchronic N_2_O exposure, several limitations should be addressed in future work. Candidate biomarkers require confirmation via targeted metabolomics using authentic standards, in larger sample sizes, and across different dose conditions. Additionally the absence of a post-exposure withdrawal group precludes determination of which metabolic changes are reversible versus persistent. Future studies incorporating a recovery period (e.g., 7–28 days) are therefore needed to distinguish transient metabolic disturbances from durable biomarkers of prior N_2_O exposure.

## Figures and Tables

**Table 1:** Differential metabolites in plasma of mice exposed to laughing gas.

Compound	VIP	FC	*p*-Value	Type
DL-tryptophan *	2.270543059	0.822417968	0.036658805	↓
His-ser *	5.39015581	0.570812231	0.002208979	↓
Ile-Pro *	3.880749431	0.459798765	0.000205238	↓
Indole *	1.517294523	0.722993645	0.005911255	↓
1-stearoyl-2-linoleoyl-sn-glycero-3-phosphoethanolamine	1.486682125	0.482374208	0.044721063	↓
Cinnamoylglycine	1.849343246	0.345705745	0.001782968	↓
Hippuric acid	1.423062144	0.421756098	0.000731456	↓
Hydroquinone	1.442359082	0.420150824	0.005596572	↓
N-acetyltryptophan	1.147914388	0.651807706	0.044526372	↓
Pc 40:7	1.129434401	1.275815744	0.012353977	↑
Pc(18:1e/9-hode)	2.393534726	0.772096354	0.049595697	↑
Pi 34:2	4.739809184	0.538354064	0.007570857	↑
Pi 36:2	6.459955298	0.458817378	0.000173552	↑
Pi 38:5	1.449694777	1.998131363	0.016594159	↑
Pi 40:6	1.137356434	0.75936702	0.033076979	↑
1-Stearoyl-2-oleoyl-sn-glycerol 3-phosphocholine (SOPC) *	6.754721173	1.303295249	0.017777616	↑
Creatine *	2.436692331	0.697139971	0.00316503	↓
Ectoine *	1.943389118	0.577586943	0.020956843	↓
PC(16:0/16:0) *	5.094645395	1.227967388	0.046586036	↑
1-heptadecanoyl-sn-glycero-3-phosphocholine	3.15992551	0.834126868	0.049949782	↓
1-o-hexadecyl-2-o-(2e-butenoyl)-sn-glyceryl-3-phosphocholine	4.527289364	1.256692825	0.035536814	↑
1-Stearoyl-sn-glycerol 3-phosphocholine	12.44580581	1.182846193	0.012812851	↑
1,2-diamino-2-methylpropane	1.18694568	0.802422286	0.033860116	↓
2-amino-1-phenylethanol	2.090094068	0.859157666	0.021316046	↓
2-aminobenzamide	1.097319299	1.623018453	0.005205103	↑
2-edahma [dmed-fahfa]	1.047672188	0.518301664	0.001830615	↓
Arg-glu	2.073750104	0.379954376	0.018220506	↓
DL-phenylalanine	1.949294435	0.840082925	0.007941554	↓
N-Docosanoyl-4-sphingenyl-1-O-phosphorylcholine	2.16056361	1.25682719	0.02727306	↑
N6-(1-iminoethyl)-l-lysine	4.474650652	1.926394568	0.027526138	↑
Polygodial	2.918300151	0.720790659	0.006298095	↓
Stachydrine	2.540478092	0.643506685	0.000893755	↓
Trigonelline	5.366086618	0.628267972	0.003109119	↓
Trimethylamine n-oxide	2.970676217	0.492690064	0.004250201	↓
Xanthosine	3.916561099	1.715063471	0.000877951	↑

Note: “↑” and “↓” indicated that the metabolites in the plasma of mice in the experimental group were up-regulated and down-regulated compared with that in the blank group. * indicates differential metabolites on significantly differential metabolic path.

## Data Availability

The original contributions presented in this study are included in the article. Further inquiries can be directed to the corresponding author.
